# RAD54B promotes gastric cancer cell migration and angiogenesis via the Wnt/β-catenin pathway

**DOI:** 10.2478/raon-2024-0007

**Published:** 2024-02-21

**Authors:** Jianchao Li, Hui Geng, Xin Li, Shenshan Zou, Xintao Xu

**Affiliations:** Department of General Surgery, Changzhou TCM Hospital, Changzhou, Jiangsu, China

**Keywords:** gastric cancer, RAD54B, migration, angiogenesis, Wnt/β-catenin

## Abstract

**Background:**

Gastric cancer is an epidemic malignancy that is commonly diagnosed at the late stage. Evidence has elucidated that RAD54B exerts a crucial role in the progress of various tumors, but its specific role and mechanism in gastric cancer remain gloomy.

**Materials and methods:**

The level of RAD54B was detected by western blot. RAD54B expression was downregulated or upregulated in both MKN45 and AGS cells by the transfection of shRAD54B or overexpression plasmid, respectively. The role of RAD54B in the growth, migration, invasion and tube formation of gastric cancer was evaluated by Edu, colony formation, transwell and tube formation assays. In addition, the molecular mechanism of RAD54B in gastric cancer was also determined by western blot. Moreover, *in vivo* experiment was conducted in xenografted mice.

**Results:**

The expression of RAD54B was discovered to be upregulated in gastric cancer based on the ATGC and GEPIA databases, which was also confirmed in gastric cancer cell lines. Moreover, overexpression of RAD54B enhanced the growth, migration, invasion, tube formation and Wnt/β-catenin signaling axis in AGS and MKN45 cells. As expected, knockdown of RAD54B in AGS and MKN45 cells reversed these promotions. More importantly, *in vivo* assay also verified that RAD54B accelerated the growth of gastric cancer and Wnt/β-catenin signaling pathway.

**Conclusions:**

Both loss-of-function and gain-of-function assays demonstrated that RAD54B facilitated gastric cancer cell progress and angiogenesis through the Wnt/β-catenin axis.

## Introduction

Gastric cancer is a category of prevalent malignancy with high invasiveness that has been demonstrated to be the fifth diagnosed cancer and fourth cause of cancer death.^1^ It has been recognized that several risk factors, including *Helicobacter pylori* infection, alcohol consumption, obesity and cigarette smoking, are strongly involved in gastric cancer.^2^ Although its incidence has been steadily decreased in the last century, a majority of gastric cancer cases are diagnosed at advanced stages nowadays.^3^ Moreover, the existence of tumor behaviors, such as metastasis and invasion forces immensely poor prognosis on the gastric cancer patients.^4^ Accordingly, the 5-year survival rate of advanced gastric cancer remains under 30% in spite enormous advance has been achieved in the therapies.^5^ Thus, discovering potential therapeutic targets and identifying the underlying molecular mechanism are of great importance for improving the gastric cancer.

RAD54 Homolog B (RAD54B) located on chromosome 8p22.1, is a member of SWI2/SNF2 heli-case superfamily.^6^ Evidence has revealed that RAD54B is associated with the homologous recombination repair and the regulation of the DNA damage checkpoint response.^7,8^ Thus, plenty of studies verify the implication between RAD54B and the progress of various cancers. For instance, upregulation of RAD54B indicates a poor survival of patients with luminal A subtype breast cancer, thus knockdown of RAD54B inhibits the growth of luminal A subtype breast cancer both *in vitro* and *in vivo*.^9^ Similarly, RAD54B is highly expressed in hepatocellular carcinoma (HCC), which has negative effects on the 5-year disease-free survival and 5-year overall survival of HCC patients. Overexpression of RAD54B greatly enhanced the cell viability and migration of HCC cells and the metastasis in xenografted mice.^10^ Also, Xu C *et al.*^11^ reported that downregulation of RAD54B attenuated the proliferation with increased apoptosis of lung cancer cells. RAD54B was highly expressed in colorectal cancer that was identified as an independent predictor of postoperative distant recurrence in patients with colorectal cancer.^12^ RAD54B is revealed to be related to the pathogenic or likely pathogenic (P/LP) germline variants in melanoma.^13^ However, the role of RAD54B in gastric cancer is still unknown.

Hence, the role and underlying molecular mechanism of RAD54B were explored in gastric cancer in the current study. We hope the results can establish an academic foundation for the development of therapeutic strategies of gastric cancer.

## Materials and methods

### Analysis of the expression profile of RAD54B in gastric cancer based on the online databases

The expression level of RAD54B in the gastric cancer samples and normal samples, as well as the pan-cancer RAD54B expression were analyzed by The Cancer Genome Atlas (TCGA). In addition, the level of RAD54B in the gastric cancer samples and normal samples was also determined by the Gene Expression Profiling Interactive Analysis (GEPIA; http://gepia2.cancer-pku.cn), an online open-access RNA-seq analysis tool^14^, in which the cutoff mRNA/transcript value and *p* value were the default values with 1 and 0.01, respectively.

### Cell culture

Human gastric cancer lines, including AGS (CL-0022), MKN45 (CL-0292), NCI-N87 (CL-0169), HGC-27 (CL-0107), human gastric epithelial cells GES-1 (CL-0563) and human umbilical vein endothelial cells (HUVECs, CL-0122) were bought from Procell (Wuhan, China). All the cell lines except for HUVECs were cultured in RPMI-1640 media (PM150110, Procell), while HUVECs were maintained in DMEM/F12 basic media (PM150312, Procell) with 10% fetal bovine serum (FBS, 1600044, Gibco, Rockville, MD, USA) and 1% penicillin/streptomycin (PB180120, Procell) at 37°C with 5% carbon dioxide (CO_2_).

### Cell transfection

Two short hairpin RNAs (shRNAs) targeting RAD54B (sh-RAD54B#1 and sh-RAD54B#2) and negative controls (sh-NC) were purchased from GenePharma (Shanghai, China). Overexpression of RAD54B was achieved via the transfection of pcDNA vector plasmids containing the sequences of RAD54B (pcDNA-RAD54B). The transfection assays were conducted with Lipofectamine 3000 (L3000015, Invitrogen, Carlsbad, CA, USA). Briefly, sh-RAD54B#1, sh-RAD54B#2, sh-NC, pcDNA-RAD54B and empty pcDNA vector plasmids (pcDNA), as well as Lipofectamine 3000 reagents were diluted with Opti-MEM™ (31985070, Invitrogen), and then mixed with a ratio of 1:1. AGS and MKN45 cells were inoculated into 6-well plates with 6×10^5^ cells per well and cultured at 37°C with 5% CO_2_. When the convergence reached 70%–90%, the mixtures were added into AGS and MKN45 cells for transfection. Cells were harvested for the subsequent examination after transfection for 48 h.

### Western blot

Total proteins from transfected AGS and MKN45 cells were isolated by RIPA buffer (R0010, Solarbio, Beijing, China) and quantified with the BCA protein quantification kit (ab102536, Abcam, Cambridge, UK) in line with the operating instructions. 20 μg protein samples were dissolved and electrically transferred onto a PVDF membrane (IPVH00010, EMD Millipore, Billerica, MA, USA). After blocking with 5% skim milk (D8340, Solarbio) at room temperature for 1 h, the membranes were incubated with primary antibodies against diverse proteins (RAD54B, 1:1000, ab168463; β-catenin, 1:500, ab16051; Axin, 1:1000, ab32197; cmyc, 1:20000, ab152146; MMP-7, 1:1000, ab216631; GAPDH, 1:2500, ab9485; all from Abcam) at 4°C overnight. Subsequently, the membranes were incubated with corresponding secondary antibodies for 2 h at room temperature and visualized by an ECL assay (P0018S, Beyotime, Shanghai, China). The band intensity was determined by ImageJ software (National Institutes of Health, USA).

**FIGURE 1.**
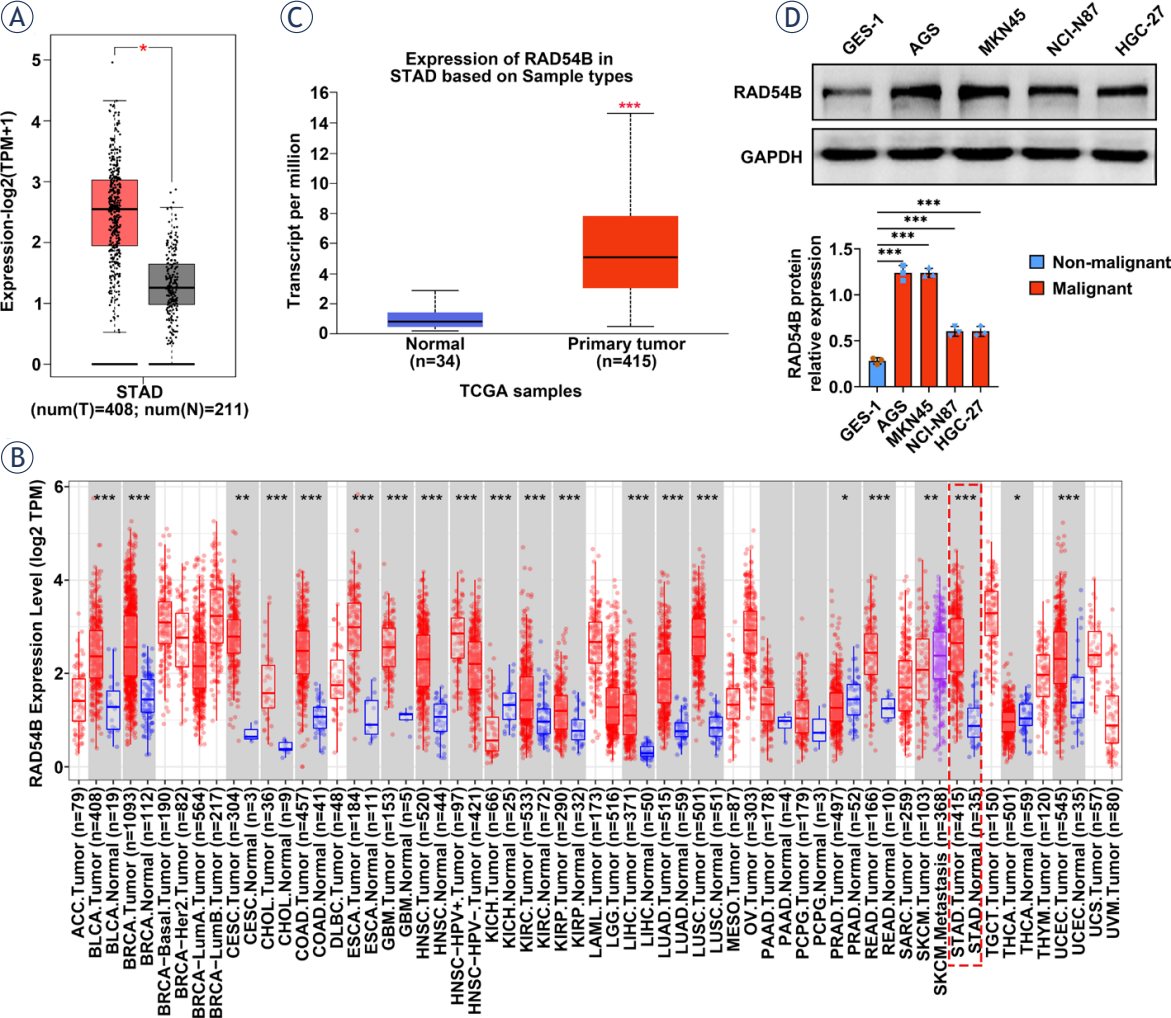
RAD54B expression was increased in gastric cancer. **(A)** The expression of RAD54B in gastric cancer was enhanced compared to normal samples according to ATGC database. **(B)** The pan-cancer analysis confirmed an increase in gastric cancer. (C) RAD54B expression was upregulated in gastric cancer based on GEPIA. (D) The expression level of RAD54B was also augmented in gastric cancer cell lines. ^*^p < 0.05; ^**^p < 0.01; ^***^p < 0.001 T = tumor tissues, N = normal tissues

### The 5-ethynyl-2′-deoxyuridine (EdU) incorporation assay

Transfected AGS and MKN45 cells with 6×10^5^ cells per well were seeded into 6-well plates and maintained at 37°C for 12 h with 5% CO_2_. Following the incubation with 1 ml of EdU working solution (20 μM) for 2 h at 37°C, cells were immobilized with immunol-staining fix solution (P0098, Beyotime), permeated with 0.3% Triton X-100 (ST795, Beyotime) and incubated with the anti-EdU Click reaction solution in dark for 30 min. Hoechst 33342 (5 μg/mL, C1022, Beyotime) was utilized for the stain of cell nucleus. The stained cells were photographed under a fluorescence microscopy (Olympus, Tokyo, Japan) and five random fields were chosen for the analysis of the EdU-positive cell percentage.

**FIGURE 2.**
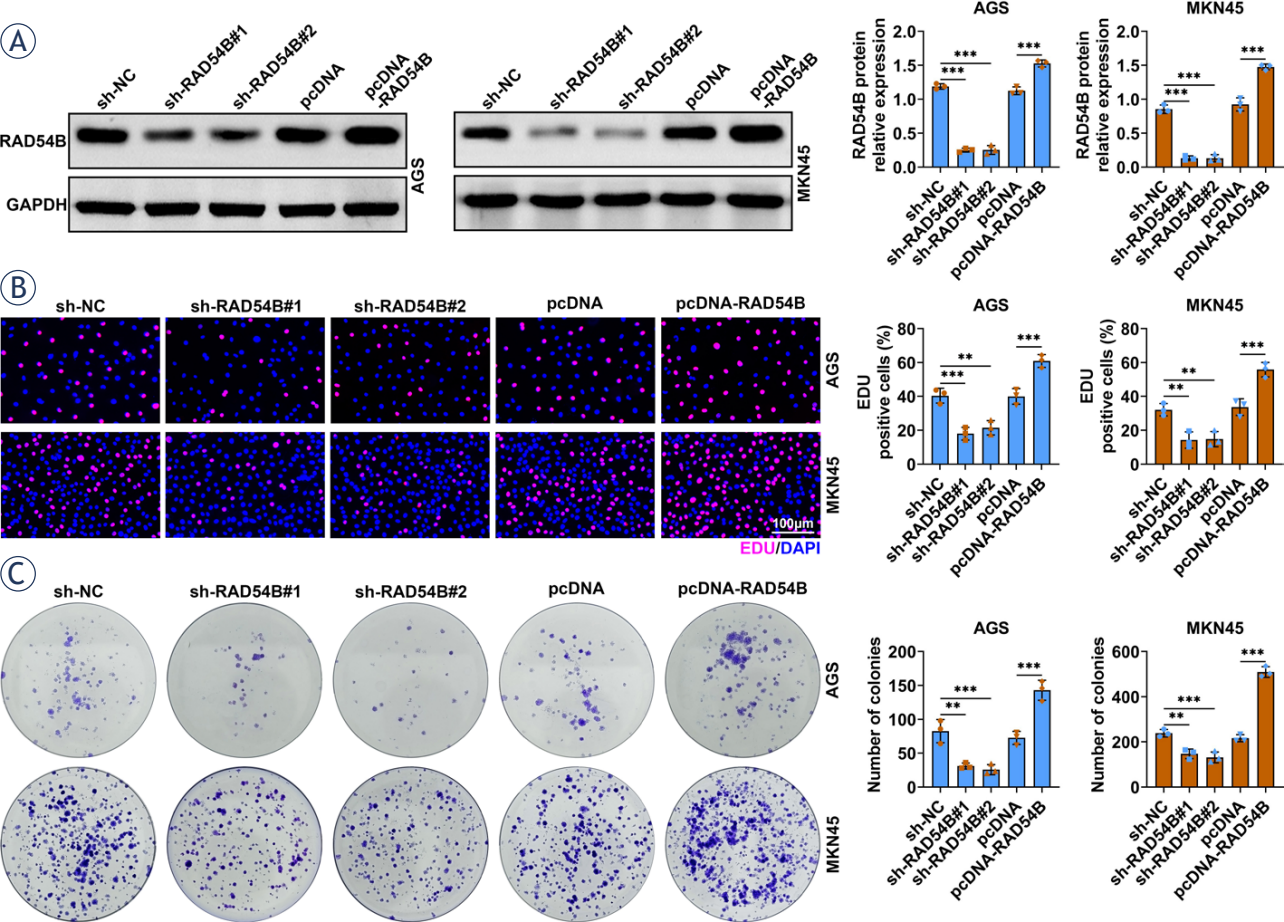
RAD54B facilitated the proliferation of gastric cancer cells. **(A)** The expression of RAD54B was downregulated or upregulated in AGS and MKN45 cells by the transfection of shRAD54B or overexpression plasmid respectively. **(B)** Transfected AGS and MKN45 cells were seeded into 6-well plates at a density of 6 × 10^5^ cells per well and maintained at 37 °C for 12 h with 5% CO_2_. The proliferation of AGS and MKN45 cells was assessed by Edu assays. **(C)** Transfected AGS and MKN45 cells with 6 × 10^5^ cells per well were plated into 6-well plates and cultured at 37°C for 14 days. The proliferation of AGS and MKN45 cells was determined by colony formation assay. ^**^p < 0.01; ^***^p < 0.001

### Colony formation assay

Transfected AGS and MKN45 cells with 6×10^5^ cells per well were plated into 6-well plates. Cells were hatched at 37°C for 14 days and then immobilized with 4% paraformaldehyde (P0099, Beyotime) and stained with 0.1% crystal violet (C0121, Beyotime) for 30 min, separately. The clone numbers were manually counted.

### Transwell assay

The mobility and invasion of transfected AGS and MKN45 cells were assessed by transwell assay using 24-well transwell chambers with 8.0-μm pore size polycarbonate membranes. Briefly, 200 μl of cell suspension with a total of 2 × 10^5^ cells and 600 μl of RPMI-1640 with 10% FBS were severally appended into the upper and lower chamber for the cell migration determination. Additionally, Matrigel matrix was filled in the transwell chamber with serum-free medium dilution for the cell invasion detection. The upper and lower chambers were diffused with 200 μl of cell suspension with a total of 2 × 10^5^ cells and 600 μl of RPMI-1640 with 10% FBS, respectively. After the continuous culture for 24 h, cells were immobilized with 4% paraformaldehyde and stained with 0.1% crystal violet for 30 min orderly, and then photographed under an inverted microscope (Olympus). Five random fields were selected for the analysis of the number of migrated and invasive cells by using the Image J software (National Institutes of Health, USA).

**FIGURE 3.**
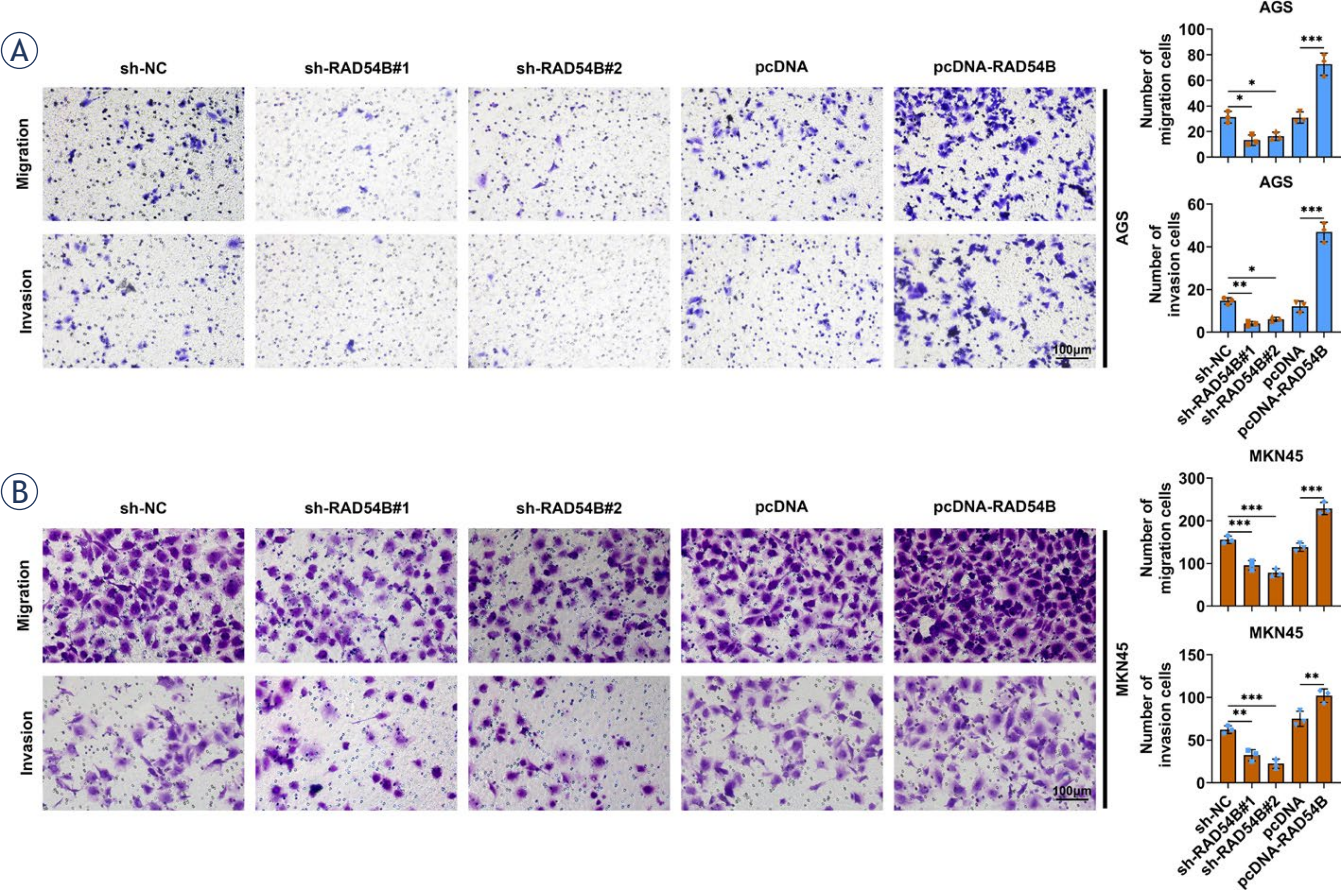
RAD54B promoted the migration and invasion of gastric cancer cells. The mobility and invasion of AGS and MKN45 cells were assessed by transwell assay using 24-well Transwell chambers with 8.0 μm pore size polycarbonate membranes. 200 μl of cells suspension with a total of 2 × 10^5^ cells was appended into the upper chamber and cultured for 24 h at 37 °C. The migration and invasion of AGS and MKN45 cells were examined by transwell assay. ^*^ *p* < 0.05; ^**^p < 0.01; ^***^p < 0.001

### Tube formation assay

sh-RAD54B#1, sh-RAD54B#2, sh-NC, pcDNA-RAD54B and empty pcDNA vector plasmids (pcDNA) were transfected into AGS and MKN45 cell lines. Supernatant was collected for the tube formation assay after transfection for 48 h. In brief, HUVECs were cultured in conditioned medium containing supernatant. Then, HUVECs were plated in a 6-well plate coated with matrigel (354248, Corning Company, New York, NY, USA) at a density of 6×10^5^ cells per well. Following 4 h, cells were examined with a phase-contrast microscopy, and the tube formation capacity was evaluated by the number of brunching points.

### Animal experiment

4 weeks-old BALB/c nude mice were bought from Vital River (Beijing, China). Mice were raised in a temperature-controlled SPF animal room with the 12-h cycle of light-dark. Ten mice were randomly divided into two groups, including sh-NC group and sh-RAD54B#1 group. Mice in both groups were subcutaneously injected with a total of 2 × 10^6^ MKN45 cells transfected with sh-NC and sh-RAD54B#1, severally. Tumor volume was monitored every seven days for continuous five weeks and calculated based on the formula: 1/2×length×width^2^. After consecutive five weeks, mice were intraperitoneally injected with 120 mg/kg sodium pentobarbital for euthanasia based on the previous study^15^, and the tumors samples were enucleated and weighed. The sh-NC group was served as a negative control. MKN45 cells transfected with sh-NC was not affecting tumor growth compared to untreated tumors that could be supported with *in vitro* results showing no differences in cell proliferation (the percent EDU positive cells and number of colonies) between control untreated and sh-NC group (Supplementary Figure 1A and B). All animal experiments were authorized by the Animal Research Ethics Committee of Changzhou TCM Hospital.

**FIGURE 4.**
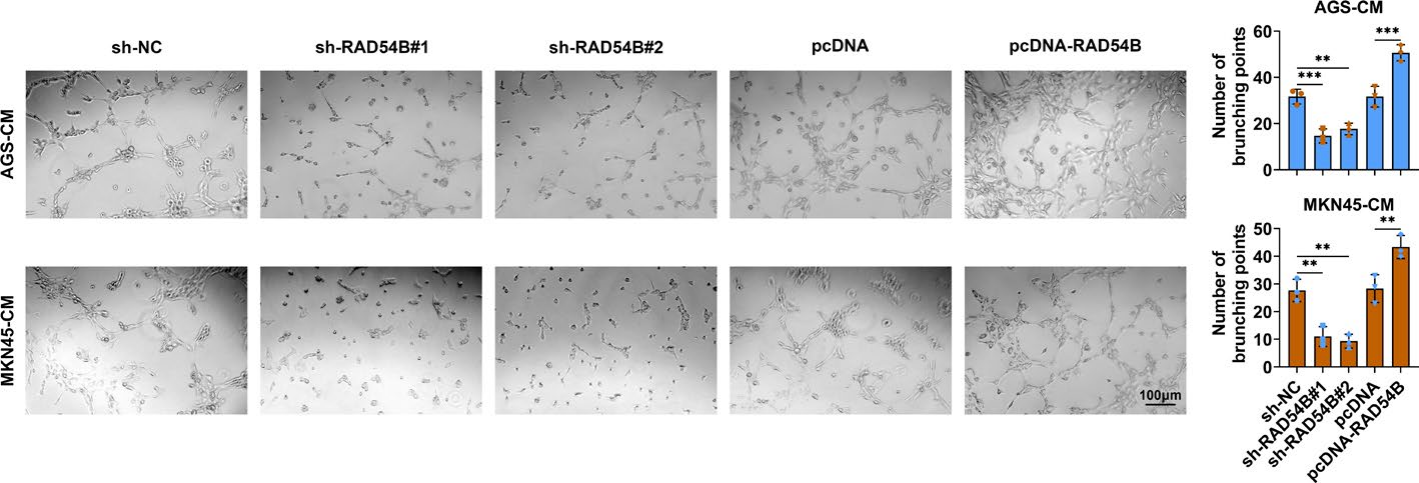
RAD54B enhanced the tube formation of gastric cancer cells. After HUVECs were inoculated into conditioned medium containing supernatant harvested from transfected AGS and MKN45 cells for co-culture, the tube formation of HUVECs was assessed by tube formation assay. ^**^p < 0.01; ^***^p < 0.001

### Immunohistochemistry (IHC)

Tumor tissues were fixed in 4% formaldehyde, dehydrated with gradient concentrations ethanol, embedded into parrafin (YA0011, Solarbio) and cut into sections with a thick of 5 μm. Sections were retrieved in 10 mM sodium citrate buffer (pH 6.0, P0083, Beyotime) for 15 min at 94°C. Following cooling to room temperature, sections were blocked with 1% bovine serum albumin (BSA, ST2249, Beyotime) for 30 min and then incubated with primary antibodies against RAD54B (1:200, ab238579, Abcam), Ki-67, c-myc (1:1000, ab32072, Abcam) and MMP-7 (1:500, ab216631, Abcam) respectively. Subsequently, sections were incubated with biotinylation-labeled secondary antibody (1:1000, ab207996, Abcam), re-stained with hematoxylin, and captured under a light microscope (Olympus). The relative level of RAD54B, Ki-67, cmyc and MMP-7 was determined as the ratio of the number of positive cells to the total number of cells.

### Statistical analysis

Three technical replicates with three independent experimental replicates were conducted in the cell experiments, while five technical replicates with three independent experimental replicates were performed in the animal experiments. Results were expressed as mean ± standard deviation (SD). Statistical differences were determined through the Student’s t-test between two groups followed by *Post Hoc* Bonferroni test by SPSS 26.0 software (IBM, Armonk, New York, USA). *P* < 0.05 was considered as significant difference.

**FIGURE 5.**
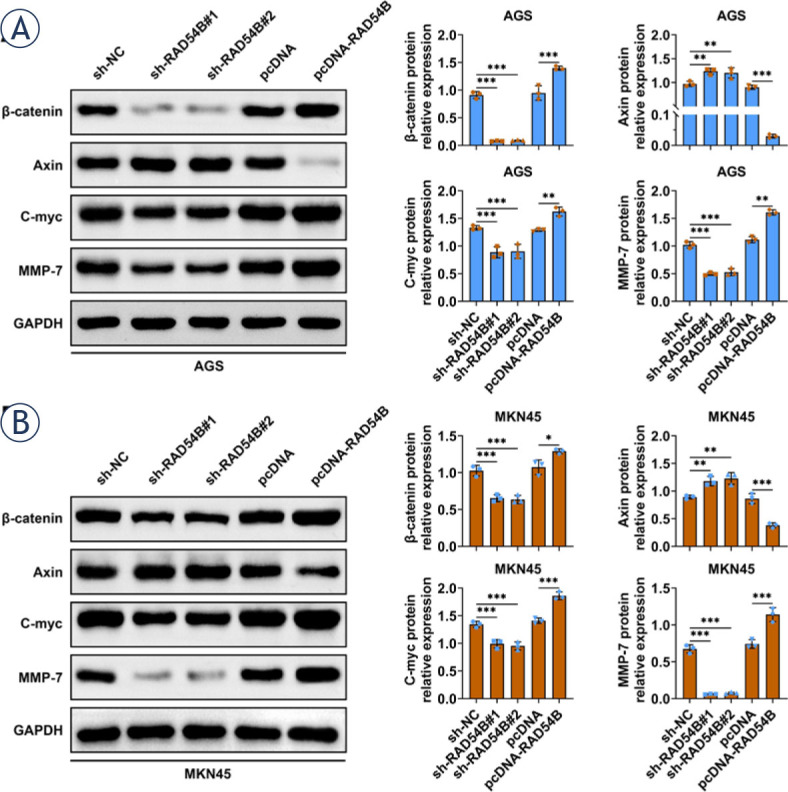
RAD54B enhanced activation of Wnt/β-catenin signaling pathway in gastric cancer cells. The relative protein expressions of β-catenin, Axin, c-myc and MMP-7 were detected by western blot. The data was expressed after being normalized to GAPDH. ^*^ *p* < 0.05; ^**^p < 0.01; ^***^p < 0.001

**FIGURE 6.**
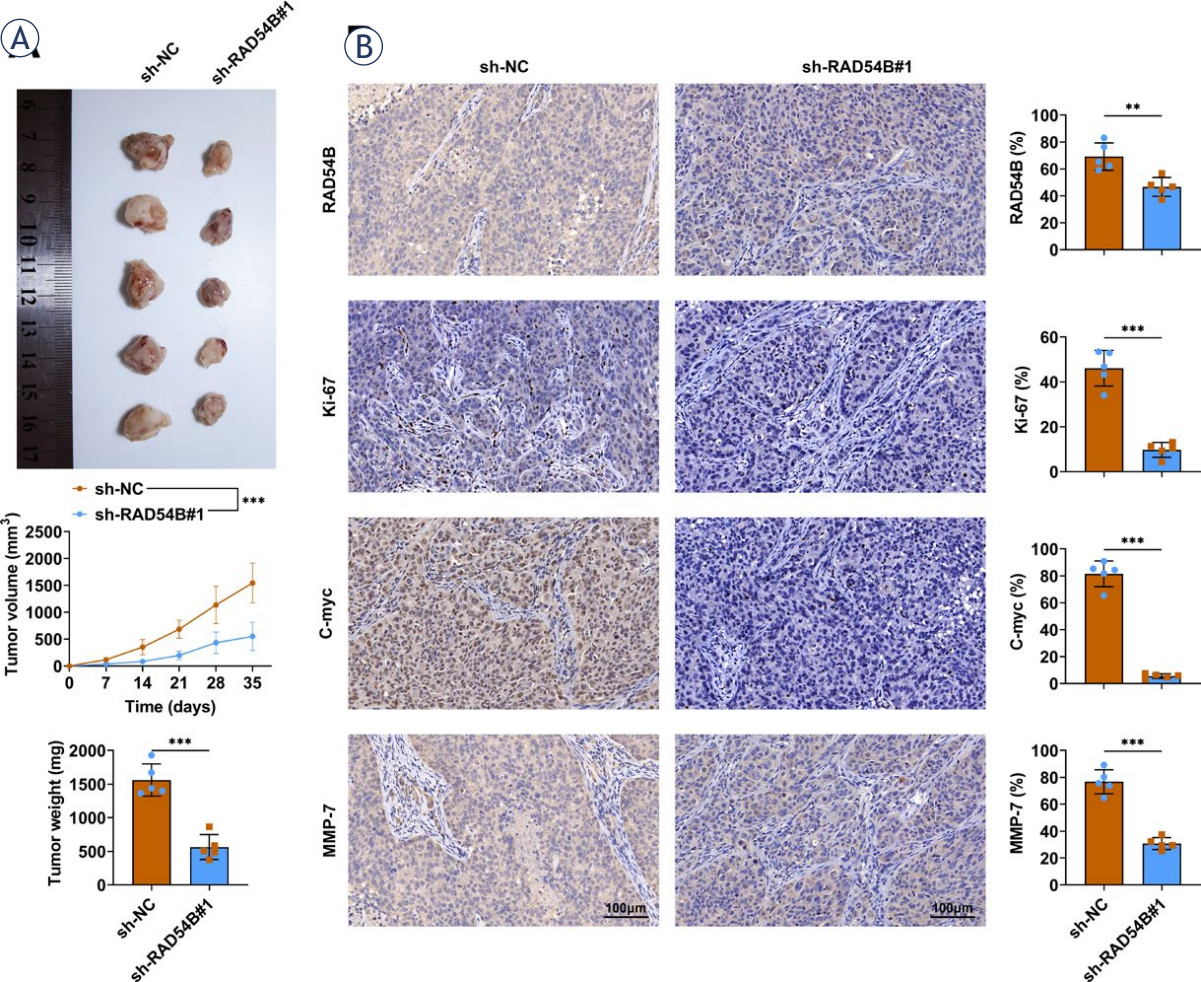
RAD54B enhanced the growth of gastric cancer and activation of Wnt/β-catenin signaling pathway *in vivo*. **(A)** Nude mice were injected with MKN45 transfected with sh-NC or sh-RAD54B#1 and then the tumor volume and weight were monitored for consecutive five weeks after the treatment. **(B)** The expression levels of RAD54B, Ki-67, c-myc and MMP-7 were determined by IHC. ^***^p < 0.001

## Results

### RAD54B was highly expressed in gastric cancer

As presented in Figure 1A, the level of RAD54B in stomach adenocarcinoma (STAD) was significantly increased compared with that in normal samples. Besides, the pan-cancer analysis revealed that RAD54B expression was also prominently enhanced in the majority of cancers, including STAD (Figure 1B). Moreover, the level of RAD54B was confirmed to be enhanced in the STAD primary tumor samples relative to that in normal samples based on the GEPIA (Figure 1C). Furthermore, a prominent increase in RAD54B expression was also observed in the gastric cancer cell lines, including AGS, MKN45, NCI-N87 and HGC-27, relative to that in human gastric epithelial cells GES-1. Among them, the expression level of RAD54B in AGS and MKN45 was significantly higher than that in NCI-N87 and HGC-27, thus the first two cell lines were chosen for subsequent assays (Figure 1D). Briefly, RAD54B expression was up-regulated in gastric cancer.

### RAD54B enhanced the growth of gastric cancer cells

Upregulated expression of RAD54B indicated that RAD54B might exert a crucial effect during the progress of gastric cancer. Hence, the expression of RAD54B was first downregulated or upregulated in both MKN45 and AGS cells with transfection of shRAD54B or overexpression plasmid respectively (Figure 2A). The Edu positive cells and numbers of colonies were observably decreased with silencing of RAD54B, while markedly increased by overexpression of RAD54B in both AGS and MKN45 cells based on the Edu (Figure 2B and C) and colony formation assays (Figure 2D and E). Moreover, no statistical difference was found in the percent EDU positive cells and number of colonies in MKN45 cells between untreated control group and sh-NC group (Supplementary Figure 1A and B). Therefore, RAD54B increased the viability of gastric cancer cells.

### RAD54B promoted the mobility and invasion of gastric cancer cells

In addition, the effect of RAD54B on the mobility and invasion of gastric cancer was also assessed by transwell assay. Both the numbers of migrated and invasive cells were notably reduced in both AGS and MKN45 cells transfected with shRAD54B (Figure 3). On the other hand, upregulation of RAD54B markedly enhanced the numbers of both migrated and invasive cells in both AGS and MKN45 cells (Figure 3). Besides, no statistical difference was found in the numbers of both migrated and invasive cells in MKN45 cells between untreated control group and sh-NC group (Supplementary Figure 1C and D). Thus, RAD54B expedited the mobility and invasion of gastric cancer cells.

### RAD54B facilitated the tube formation of gastric cancer cells

Moreover, supernatants form cultured AGS and MKN45 cells with the knockdown of RAD54B or with the overexpression of RAD54B were used to incubate with HUVEC. The results showed that the number of brunching points of HUVECs was significantly diminished after HUVECs were cultured with supernatants form cultured AGS and MKN45 cells with the knockdown of RAD54B, while that was prominently increased after HUVECs were cultured with supernatants form cultured AGS and MKN45 cells with the overexpression of RAD54B (Figure 4), indicating that RAD54B promoted the tube formation of gastric cancer cells.

### RAD54B activated Wnt/β-catenin signaling axis in gastric cancer cells

To explore the molecular mechanism of RAD54B in the progress of gastric cancer, the relative protein levels of β-catenin, Axin, c-myc and MMP-7 were examined via western blot. As displayed in Figure 5, the relative protein expressions of β-catenin, c-myc and MMP-7 were significantly decreased in both AGS and MKN45 cells with transfection of shRAD54B, while notably augmented in both AGS and MKN45 cells transfected with RAD54B overexpression plasmid. On the contrary, downregulation of RAD54B markedly enhanced the relative protein expression of Axin, whereas upregulation of RAD54B observably decreased the relative protein expression of Axin in both AGS and MKN45 cells. Therefore, these results manifested that RAD54B facilitated the activation of Wnt/β-catenin signaling axis in gastric cancer cells.

### RAD54B accelerated the growth of gastric cancer *in vivo*

To further verify the role of RAD54B in gastric cancer, nude mice were injected with MKN45 transfected with sh-NC or sh-RAD54B#1 and then monitored for sequential five weeks. As shown in Figure 6A, results exhibited that knockdown of RAD54B significantly decreased the tumor volume and weight (Figure 6A). In addition, silencing of RAD54B also prominently reduced the expression levels of RAD54B, Ki-67, c-myc and MMP-7 relative to sh-NC group (Figure 6B). Thus, these outcomes demonstrated that RAD54B accelerated the proliferation of gastric cancer and activation of Wnt/β-catenin signaling axis *in vivo*.

## Discussion

Gastric cancer is an epidemic malignancy, which is always diagnosed at the late stage.^3^ Thus, despite the advance and survival of gastric cancer have been prominently increased in recent decades, the prognosis remains discontented duo to its high recurrence rate.^16^ Evidence has elaborated that RAD54B exerts a significant role in a variety of cancers^17^, but its specific role and mechanism in gastric cancer are still unknown. In the current study, the level of RAD54B was discovered to be enhanced in gastric cancer based on the ATGC and GEPIA databases, which was also confirmed in gastric cancer cell lines. Moreover, overexpression of RAD54B enhanced the growth, migration, invasion, tube formation and activation of Wnt/β-catenin signaling axis in AGS and MKN45 cells, which was reversed by knockdown of RAD54B in AGS and MKN45 cells. More importantly, *in vivo* assay also verified that RAD54B accelerated the growth of gastric cancer and activation of Wnt/β-catenin signaling pathway. Based on these outcomes, we concluded that RAD54B facilitated gastric cancer cell progress and angiogenesis through activating the Wnt/β-catenin pathway.

High expression of RAD54B has been founded in a series of cancers, such as breast cancer and its subtype^9,18^, HCC^10,19^, lung cancer^11^, and colorectal cancer^12^, which has been verified in our pan-cancer analysis, as indicated by a remarkable increase of RAD54B expression in BRCA, LIHC, LUAD, LUSC, COAD and READ. In line with these findings, RAD54B expression was also upregulated in gastric cancer according to the ATGC and GEPIA databases, which has been confirmed in gastric cancer cell lines as well. Zhang Z *et al.*^9^ demonstrated that enrichment of RAD54B in luminal A breast cancer promoted tumor cell proliferation, apoptosis and cell cycle arrrest. Feng S *et al.*^10^ validated that high-expression of RAD54B facilitated the growth and mobility of HCC cells, as well as the metastasis ability *in vivo*. Xu C *et al.*^11^ reported that upregulation of RAD54B enhanced lung cancer signatures involved in proliferation and apoptosis. In the current study, overexpression of RAD54B consistently promoted the proliferation, migration and invasion of both AGS and MKN45 cells through gain-of-function examination, *vice versa*. Moreover, the suppressive effect of shRAD54B on the tumor volume and weight was also verified in xenografted mice. Thus, these findings clarified that RAD54B expression was notably enhanced in gastric cancer, which accelerated the growth, mobility and invasion of gastric cancer cells.

Angiogenesis is a pivotal process involved in the blood vessels formation that follows a sequence of serial steps for vascular branching.^20^ Angiogenesis is required for the growth, development, wound healing and regeneration, thus it is generally associated with various physiological and pathological processes.^21^ A growing number of studies has \demonstrated that angiogenesis is implicated with tumorigenesis, in which angiogenesis is imperative for the tumor proliferation and metastasis through the nutrient supply.^21,22^ Hence, strategies targeting anti-angiogenesis are significant for cancer treatment. Here, downregulation of RAD54B notably reduced the number of brunching points, and upregulation of RAD54B prominently enhanced the number of brunching points of both AGS and MKN45 cells. Therefore, both loss-of-function and gain-of-function assays expounded that RAD54B promoted the tube formation of gastric cancer cells. However, the method used in the present study for the detection of angiogenesis was only the tube formation assay, which may lead to potential bias or imprecision. Thus, more abundant methods should be utilized to assess the role of RAD54B in the angiogenesis in the future.

Mechanically, RAD54B facilitated activation of Wnt/β-catenin signaling pathway both *in vitro* and *in vivo*. Wnt/β-catenin signaling axis is associated with a variety of physiological processes, such as embryonic development, regeneration, growth and homeostasis, hence unusual activation Wnt/β-catenin signaling is always observed in the different cancers.^23^ Extensive studies have reported that Wnt/β-catenin signaling is associated with the progress of diverse cancers. For instance, IFIT1 activates Wnt/β-catenin signaling in pancreatic cancer to enhance its growth, mobility and invasion.^24^ Inhibin subunit beta A modulates the proliferation, migration and invasion via activation of Wnt/β-catenin signaling in breast cancer.^25^ Inhibition of Wnt/β-catenin pathway through C644-0303 also impedes the growth of colorectal cancer.^26^ In the current study, the expression levels of β-catenin, c-myc and MMP-7 were notably diminished in both AGS and MKN45 cells with transfection of shRAD54B and notably increased in both AGS and MKN45 cells transfected with RAD54B over-expression plasmid, which also confirmed *in vivo*. However, the contrary results were obtained in the relative protein expression of Axin in both AGS and MKN45 cells. Axin is a dominating constituent of the canonical Wnt signaling axis, and it contains functional domains that bind to many members involved in the Wnt signaling axis, such as adenomatous polyposis coli (APC), glycogen synthase kinase 3β (GSK3β) and β-catenin.^27^ Axin owns a dual role in regulating Wnt signaling.^27^ On the one hand, Axin, as a scaffold protein with multiple domains, can form a β-catenin destruction complex (APC-Axin-GSK-3β), which facilitates the degradation of β-catenin, and effectively regulates β-catenin to maintain a very low concentration in normal cells, thereby inhibiting Wnt signaling. Additionally, Axin interacts with low-density lipoprotein receptor-related proteins 5 or 6 (LRP5/6) and promotes the recruitment of GSK3 to the plasma membrane to enhance LRP5/6 phosphorylation and activation of Wnt signaling. Accumulated β-catenin within the nucleus can interact with T-cell factor-lymphoid enhancer-binding factor (Tcf-Lef) to enable Wnt-respondent gene transcription, containing c-Myc and Cyclin D1, eventually causing the alterations in the proliferation.^23^ MMP-7 is transcriptionally modulated by β-catenin and also one of primary downstream regulator of canonical Wnt signaling reported in diverse cancers, such as ovarian endometrial carcinoma^28^, intestinal adenoma^29^ and colorectal cancer.30 Moreover, it has been demonstrated that RAD54B facilitated the progression of HCC via regulating the Wnt/β-catenin signaling axis.^10^ Altogether, these findings elaborated that RAD54B facilitated gastric cancer progression and angiogenesis through activating the Wnt/β-catenin axis. Nevertheless, a direct connection between the Wnt/β-catenin signaling and the progression of gastric cancer should be validated in the subsequent studies through the pharmacological block or other effective interference.

In summary, the results in the current study clarified that RAD54B level was significantly upregulated in gastric cancer. Both loss-of-function and gain-of-function assays illustrated that RAD54B promoted the growth, mobility, invasion and tube formation of gastric cancer. Mechanically, RAD54B activated Wnt/β-catenin signaling. Therefore, RAD54B accelerated gastric cancer progress and angiogenesis by activating the Wnt/β-catenin pathway. Briefly, our findings can lay a theoretical basic for the development of diagnosis biomarker and therapy target for gastric cancer treatment.

## Supplementary Material

Supplementary Material Details
