# Supplementary material for: RAD54B promotes gastric cancer cell migration and angiogenesis via the Wnt/β-catenin pathway
Source: Radiol Oncol. 2024 Feb 21;58(1):67–77. doi: 10.2478/raon-2024-0007 (PMC10878776; doi:10.2478/raon-2024-0007)
Supplement: Supplementary file 1 — Supplementary Material Details [file raon-2024-0007-sm.pdf]

RAD54B promotes gastric cancer cell migration and angiogenesis via the Wnt/β-catenin pathway

Jianchao Li, Hui Geng, Xin Li, Shenshan Zou, Xintao Xu

Radiol Oncol 2024; 58(1): 67-77.

doi: 10.2478/raon-2024-0007

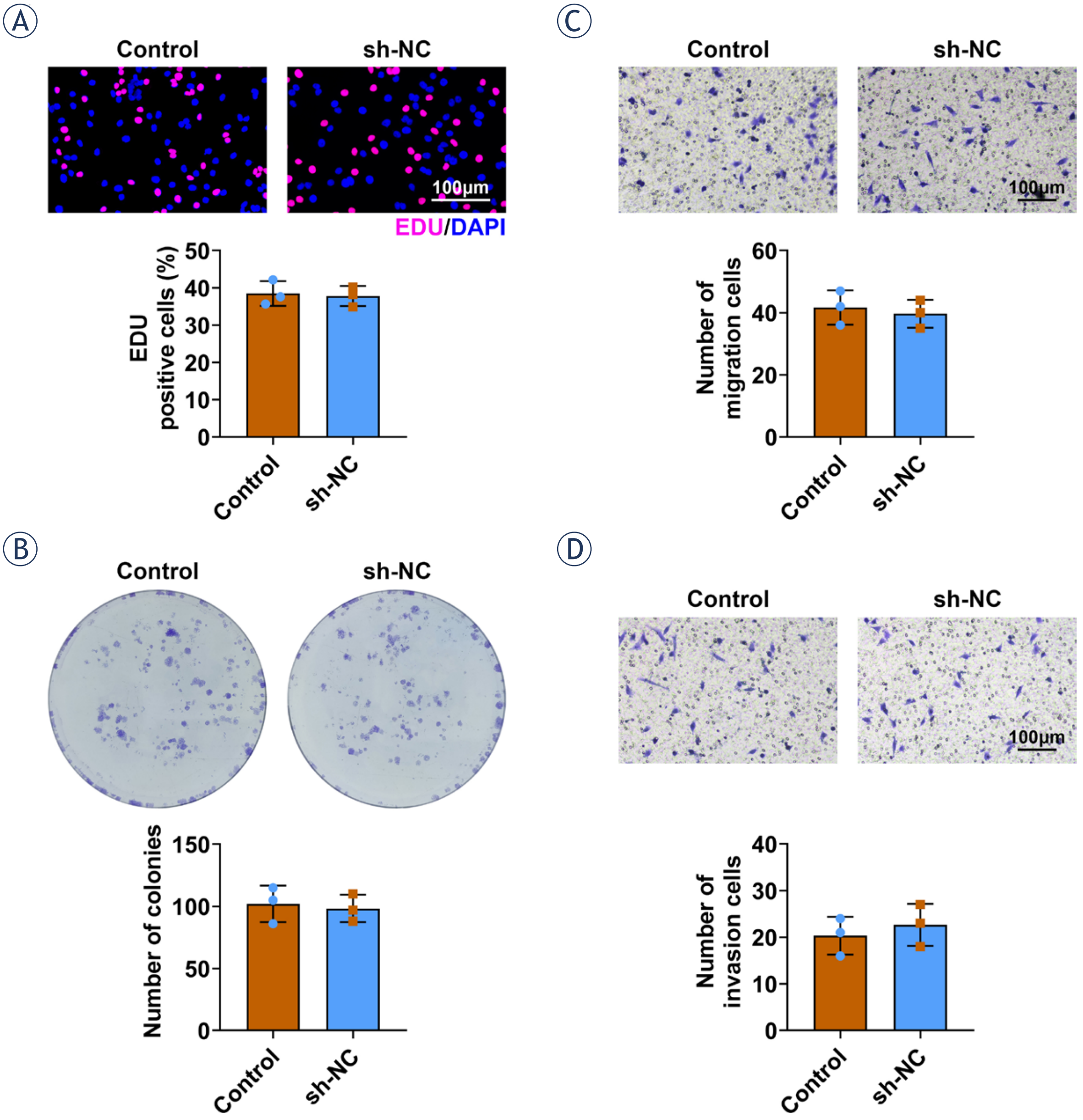

SUPPLEMENTARY FIGURE 1. Comparison the proliferation, migration and invasion in MKN45 cells without treatment or transfected with sh-NC. **(A)** The proliferation MKN45 cells was evaluated by Edu assays. **(B)** The proliferation of MKN45 cells was examined by colony formation assay. **(C)** The mobility of MKN45 cells were assessed by transwell assay. **(D)** The invasion of MKN45 cells were determined by transwell assay.
